# Supplementary material for: Urban–Rural Disparities in Community Participation after Spinal Cord Injury in Ontario
Source: Healthcare (Basel). 2024 Oct 20;12(20):2089. doi: 10.3390/healthcare12202089 (PMC11507304; doi:10.3390/healthcare12202089)
Supplement: Supplementary file 1 [file healthcare-12-02089-s001.zip › healthcare-3210170-supplementary.pdf]

Community Participation

SCI-HIGH

In this survey, we intend to measure self-efficacy in performing functional activities of daily living in individuals with spinal cord injury using the Moorong Self-Efficacy Scale (MSES) and to assess the individual's satisfaction with performance in life activities using the Reintegration to Normal Living Index (RNLI). This information will be recorded using the following multiple-choice survey and will be used to inform the care and education provided during and after spinal cord injury rehabilitation.

The personal information record in this survey will be only seen by Dr. Cathy Craven (project leader) and SCI-High team members. Other project members may see a summary of the information, without your personal information. Data will be reported in aggregate form, and you will not be named in any reports or publications now or in the future. The project leader will keep your answers anonymous (without your name or other information that can identify you) over the next three years. All digital information will be stored in a secure and encrypted hospital server.

PARTICIPANT'S INFORMATION

Reachlite ID: ___________________

Consortium ID: _____________________________

Please, use a dark pen to mark the squares with "Xs" (☒) *or* checkmarks (☑).

1. **Do you self-identify as a:**

☐ Female ☐ Male ☐ Another gender identity ☐ Prefer not to say

2. **What are the first three digits of your current postal code?**

First digit	*Second digit*	*Third digit*
A☐ B☐ C☐ D☐ E☐ F☐ G☐	1☐ 2☐ 3☐	A☐ B☐ C☐ D☐ E☐ F☐ G☐
H☐ I☐ J☐ K☐ L☐ M☐ N☐	4☐ 5☐ 6☐	H☐ I☐ J☐ K☐ L☐ M☐ N☐
O☐ P☐ Q☐ R☐ S☐ T☐ U☐	7☐ 8☐ 9☐	O☐ P☐ Q☐ R☐ S☐ T☐ U☐
V☐ W☐ X☐ Y☐ Z☐	0☐	V☐ W☐ X☐ Y☐ Z☐

5. What year were you born?

☐ 1930	☐ 1940	☐ 1950	☐ 1960	☐ 1970	☐ 1980	☐ 1990	☐ 2000
☐ 1931	☐ 1941	☐ 1951	☐ 1961	☐ 1971	☐ 1981	☐ 1991	☐ 2001
☐ 1932	☐ 1942	☐ 1952	☐ 1962	☐ 1972	☐ 1982	☐ 1992	☐ 2002
☐ 1933	☐ 1943	☐ 1953	☐ 1963	☐ 1973	☐ 1983	☐ 1993	☐ 2003
☐ 1934	☐ 1944	☐ 1954	☐ 1964	☐ 1974	☐ 1984	☐ 1994	☐ 2004
☐ 1935	☐ 1945	☐ 1955	☐ 1965	☐ 1975	☐ 1985	☐ 1995	☐ 2005
☐ 1936	☐ 1946	☐ 1956	☐ 1966	☐ 1976	☐ 1986	☐ 1996	☐ 2006
☐ 1937	☐ 1947	☐ 1957	☐ 1967	☐ 1977	☐ 1987	☐ 1997	☐ 2007
☐ 1938	☐ 1948	☐ 1958	☐ 1968	☐ 1978	☐ 1988	☐ 1998	☐ 2008
☐ 1939	☐ 1949	☐ 1959	☐ 1969	☐ 1979	☐ 1989	☐ 1999	☐ 2009

6. How long ago did you had your spinal cord injury/disease?

☐ 0-6 months	☐ 2-5 years	☐ 20-25 years	☐ >40 years
☐ 6-12 months	☐ 6-10 years	☐ 25-30 years	☐ Don't know
☐ 12-18 months	☐ 10-15 years	☐ 30-35 years	
☐ 18-24 months	☐ 15-20 years	☐ 35-40 years	

7. What was the cause of your spinal cord injury/disease? (pick one)

☐ Trauma (fall, car accident, etc.) ☐ Nontrauma (disease or surgery)

8. Do you have: (*pick one*) ☐ Paraplegia ☐ Quadriplegia

9. Do you have motor function below the level of injury? ☐ Yes ☐ No

10. Do you have pinprick or light touch sensation below the level of injury? ☐ Yes ☐ No

11. Most of the time, what mobility device do you use <u>outside</u> your home? (*pick one*)

☐ Manual wheelchair	☐ Walker	☐ Walking without assistance
☐ Power wheelchair	☐ Cane	☐ Other

12. Most of the time, what mobility device do you use <u>inside</u> your home? (*pick one*)

☐ Manual wheelchair	☐ Walker	☐ Walking without assistance
☐ Power wheelchair	☐ Cane	☐ Other

 December, 2021 ©SCI-High Project/UHN

Moorong Self-Efficacy Scale (MSES)
The MSES questionnaire was developed to measure self-efficacy in performing functional activities of daily living in individuals with SCI. It consists of two factors: daily activities (e.g., I can maintain my personal hygiene with or without help), and social functioning (e.g., I can enjoy spending time with my friends). The MSES is a self-report questionnaire and participants rate their confidence in their ability to complete the 16 tasks on a seven-point Likert scale (1: Very uncertain, 7: Very certain). Approximately 5 minutes is required to administer the test.

Remember, this questionnaire is not asking whether or not you have been doing these things, but rather how certain you are that you could do them.

1. I can maintain my person hygiene with or without help.

   ☐ 1 ☐ 2 ☐ 3 ☐ 4 ☐ 5 ☐ 6 ☐ 7

   Very uncertain Very certain

2. I can avoid having bowel accidents.

   ☐ 1 ☐ 2 ☐ 3 ☐ 4 ☐ 5 ☐ 6 ☐ 7

   Very uncertain Very certain

3. I can participate as an active member of the household.

   ☐ 1 ☐ 2 ☐ 3 ☐ 4 ☐ 5 ☐ 6 ☐ 7

   Very uncertain Very certain

4. I can maintain relationships in my family.

   ☐ 1 ☐ 2 ☐ 3 ☐ 4 ☐ 5 ☐ 6 ☐ 7

   Very uncertain Very certain

5. I can get out of my house whenever I need to.

   ☐ 1 ☐ 2 ☐ 3 ☐ 4 ☐ 5 ☐ 6 ☐ 7

   Very uncertain Very certain

December, 2021 ©SCI-High Project/UHN

6. I can have a satisfying sexual relationship.

☐ 1 ☐ 2 ☐ 3 ☐ 4 ☐ 5 ☐ 6 ☐ 7

Very uncertain Very certain

7. I can enjoy spending time with my friends.

☐ 1 ☐ 2 ☐ 3 ☐ 4 ☐ 5 ☐ 6 ☐ 7

Very uncertain Very certain

8. I can find hobbies and leisure pursuits that interest me.

☐ 1 ☐ 2 ☐ 3 ☐ 4 ☐ 5 ☐ 6 ☐ 7

Very uncertain Very certain

9. I can maintain contact with people who are important to me.

☐ 1 ☐ 2 ☐ 3 ☐ 4 ☐ 5 ☐ 6 ☐ 7

Very uncertain Very certain

10. I can deal with unexpected problems that come up in life.

☐ 1 ☐ 2 ☐ 3 ☐ 4 ☐ 5 ☐ 6 ☐ 7

Very uncertain Very certain

11. I can imagine being able to work at some time in the future.

☐ 1 ☐ 2 ☐ 3 ☐ 4 ☐ 5 ☐ 6 ☐ 7

Very uncertain Very certain

12. I can accomplish most things I set out to do.

☐ 1 ☐ 2 ☐ 3 ☐ 4 ☐ 5 ☐ 6 ☐ 7

Very uncertain Very certain

December, 2021 ©SCI-High Project/UHN

13. When trying to learn something new, I will persist until I am successful.

☐ 1 ☐ 2 ☐ 3 ☐ 4 ☐ 5 ☐ 6 ☐ 7

Very uncertain Very certain

14. When I see someone I would like to meet, I am able to make the first contact.

☐ 1 ☐ 2 ☐ 3 ☐ 4 ☐ 5 ☐ 6 ☐ 7

Very uncertain Very certain

15. I can maintain good health and wellbeing.

☐ 1 ☐ 2 ☐ 3 ☐ 4 ☐ 5 ☐ 6 ☐ 7

Very uncertain Very certain

16. I can imagine having a fulfilling lifestyle in the future.

☐ 1 ☐ 2 ☐ 3 ☐ 4 ☐ 5 ☐ 6 ☐ 7

Very uncertain Very certain

Reintegration to Normal Living Index (RNLI)

The Reintegration to Normal Living Index (RNLI) is a self-report questionnaire that assesses an individual's satisfaction with performance in life activities. It assesses mobility, self-care, daily activity, recreational activity, and family roles. It was developed based on interviews with clinicians, patients and their significant others. Wheelchairs or other assistive/adaptive aids may be used when considering how to answer the questions. Administration time is approximately 10 minutes.
There are 11 items in the RNLI questionnaire. Each of these items is accompanied by a visual analog scale (VAS) anchored by phrases reflecting whether the statement describes the situation of the patient. It allows the patients to determine the extent to which the statement in question applies to their specific situation. Each VAS is scored out of 10 points: 1 = minimal reintegration, 10 = complete reintegration.

1. I move around my living quarters as I feel is necessary. (Wheelchairs, other equipment or resources may be used.)

☐ 1 ☐ 2 ☐ 3 ☐ 4 ☐ 5 ☐ 6 ☐ 7 ☐ 8 ☐ 9 ☐ 10

no reintegration complete reintegration

 December, 2021 ©SCI-High Project/UHN

2. I move around my community as I feel is necessary. (Wheelchairs, other equipment or resources may be used.)

☐ 1 ☐ 2 ☐ 3 ☐ 4 ☐ 5 ☐ 6 ☐ 7 ☐ 8 ☐ 9 ☐ 10
no reintegration — complete reintegration

3. I am able to take trips out of town as I feel are necessary. (Wheelchairs, other equipment or resources may be used.)

☐ 1 ☐ 2 ☐ 3 ☐ 4 ☐ 5 ☐ 6 ☐ 7 ☐ 8 ☐ 9 ☐ 10
no reintegration — complete reintegration

4. I am comfortable with how my self-care needs (dressing, feeding, toileting, bathing) are met. (Adaptive equipment, supervision and/or assistance may be used.)

☐ 1 ☐ 2 ☐ 3 ☐ 4 ☐ 5 ☐ 6 ☐ 7 ☐ 8 ☐ 9 ☐ 10
no reintegration — complete reintegration

5. I spend most of my days occupied in a work activity that is necessary or important to me. (Work activity could be paid employment, housework, volunteer work, school, etc. Adaptive equipment, supervision and/or assistance may be used.)

☐ 1 ☐ 2 ☐ 3 ☐ 4 ☐ 5 ☐ 6 ☐ 7 ☐ 8 ☐ 9 ☐ 10
no reintegration — complete reintegration

6. I am able to participate in recreational activities (hobbies, crafts, sports, reading, television, games, computers, etc.) as I want to. (Adaptive equipment, supervision and/or assistance may be used.)

☐ 1 ☐ 2 ☐ 3 ☐ 4 ☐ 5 ☐ 6 ☐ 7 ☐ 8 ☐ 9 ☐ 10
no reintegration — complete reintegration

7. I participate in social activities with family, friends, and/or business acquaintances as is necessary or desirable to me. (Adaptive equipment, supervision and/or assistance may be used.)

☐ 1 ☐ 2 ☐ 3 ☐ 4 ☐ 5 ☐ 6 ☐ 7 ☐ 8 ☐ 9 ☐ 10
no reintegration — complete reintegration

December, 2021 ©SCI-High Project/UHN

8. I assume a role in my family which meets my needs and those of other family members. (Family means people with whom you live and/or relatives with whom you don't live but see on a regular basis. Adaptive equipment, supervision and/or assistance may be used.)

☐ 1	☐ 2	☐ 3	☐ 4	☐ 5	☐ 6	☐ 7	☐ 8	☐ 9	☐ 10
no reintegration									complete reintegration

9. I assume a role in my family which meets my needs and those of other family members. (Family means people with whom you live and/or relatives with whom you don't live but see on a regular basis. Adaptive equipment, supervision and/or assistance may be used.)

☐ 1	☐ 2	☐ 3	☐ 4	☐ 5	☐ 6	☐ 7	☐ 8	☐ 9	☐ 10
no reintegration									complete reintegration

10. In general, I am comfortable with my personal relationships.

☐ 1	☐ 2	☐ 3	☐ 4	☐ 5	☐ 6	☐ 7	☐ 8	☐ 9	☐ 10
no reintegration									complete reintegration

11. In general, I am comfortable with myself when I am in the company of others.

☐ 1	☐ 2	☐ 3	☐ 4	☐ 5	☐ 6	☐ 7	☐ 8	☐ 9	☐ 10
no reintegration									complete reintegration

12. I feel that I can deal with life events as they happen.

☐ 1	☐ 2	☐ 3	☐ 4	☐ 5	☐ 6	☐ 7	☐ 8	☐ 9	☐ 10
no reintegration									complete reintegration

December, 2021 ©SCI-High Project/UHN
